# Supplementary material for: Preparation of Biocompatible Manganese Selenium-Based Nanoparticles with Antioxidant and Catalytic Functions
Source: Molecules. 2023 Jun 1;28(11):4498. doi: 10.3390/molecules28114498 (PMC10254917; doi:10.3390/molecules28114498)
Supplement: Supplementary file 1 [file molecules-28-04498-s001.zip › molecules-2347358-supplementary.pdf]

Supporting Information

Supplementary figures

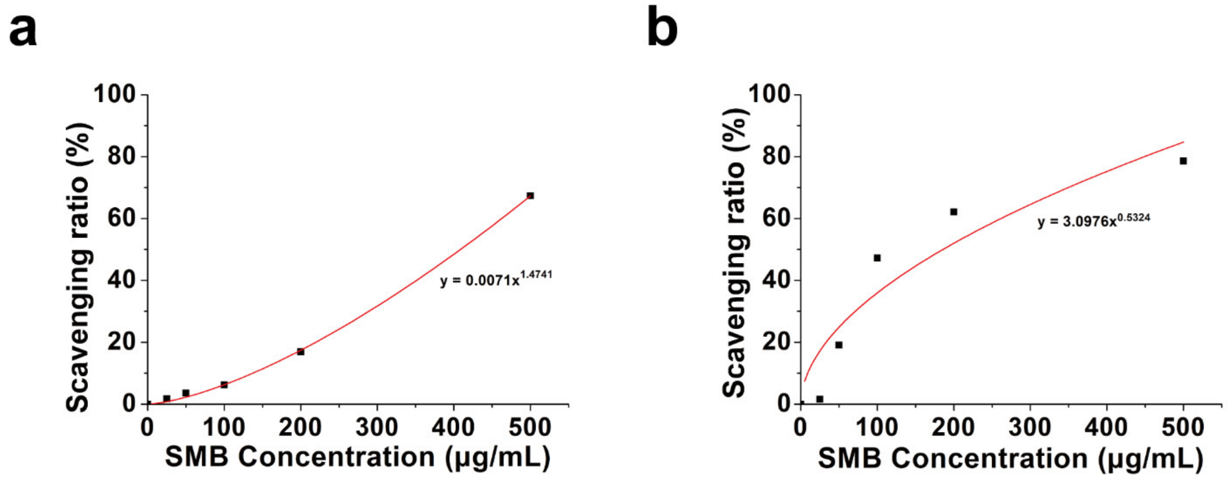

Figure S1. a) Fitted graphs of SMB NPs on $ABTS^{+}$ scavenging ratio. b) Fitted graphs of SMB NPs on DPPH scavenging ratio.
